# Aberration correction and complementary beam subtraction in light-sheet fluorescence microscopy

**DOI:** 10.1117/1.JBO.31.9.096501

**Published:** 2026-09-22

**Authors:** Yang Zhang, Ruiwen Yang, Xing Li, Dan Dan, Yanlong Yang, Yuzhu Liu, Runze Li, Jia Qian, Taiqiang Dai, Liang Kong, Xianghua Yu, Baoli Yao

**Affiliations:** a Chinese Academy of Sciences, State Key Laboratory of Ultrafast Optical Science and Technology, Xi’an Institute of Optics and Precision Mechanics, Xi’an, China; b University of Chinese Academy of Sciences, Beijing, China; c The Fourth Military Medical University, State Key Laboratory of Oral & Maxillofacial Reconstruction and Regeneration, National Clinical Research Center for Oral Diseases, Department of Oral and Maxillofacial Surgery, School of Stomatology, Xi’an, China

**Keywords:** light-sheet fluorescence microscopy, complementary beam subtraction, adaptive optics, aberration correction

## Abstract

**Significance:**

Scanning nondiffracting Bessel beams in light-sheet fluorescence microscopy (LSFM) can expand the field of view (FOV), but side lobes of the Bessel beams produce the out-of-focus background and limit the axial resolution. Furthermore, optical aberrations degrade the image quality and affect the imaging depth.

**Aim:**

To compensate wavefront distortions and enhance the imaging performance in LSFM, we propose a method that combines the adaptive optics (AO) with the complementary beam subtraction (CBS) method in LSFM, referred as AO-CBS.

**Approach:**

In the AO-CBS method, the sensorless AO is employed to correct optical aberrations in LSFM, resulting in a notable enhancement of image contrast and signal intensity by 48%. Higher axial resolution is achieved by effectively removing the out-of-focus background through subtracting the two image stacks acquired by scanning the Bessel beam and its corresponding complementary beam.

**Results:**

The final axial resolution is improved from 2.13±0.18  μm to 1.19±0.02  μm with AO-CBS methods. The effectiveness of AO-CBS in correcting aberrations is demonstrated through imaging of fluorescent beads, *Aspergillus* conidia, cleared mouse liver section, and other biological tissues.

**Conclusions:**

The AO-CBS method effectively addresses key issues existing in traditional Bessel beam-based LSFM, including the sidelobe-induced out-of-focus background, the limited axial resolution, and the degraded image quality caused by optical aberrations. The successful application of this idea in LSFM can serve as a design strategy to enhance the light-sheet-based imaging systems, thereby promoting the development and the application of LSFM and providing more powerful tools for biomedical imaging.

## 1 Introduction

Advanced optical microscopy is indispensable for visualizing and quantifying microscopic dynamic processes in living biological specimens. Such microscopy must exhibit minimal photodamage while providing fast, high temporal-spatial resolution three-dimensional imaging over a wide field of view (FOV). Light-sheet fluorescence microscopy (LSFM) illuminates specimens with a thin light sheet (LS) from the side and captures images orthogonally, inherently possessing optical section property by effectively avoiding the generation of the out-of-focus background.^1^^–^^3^ The selective illumination strategy significantly reduces photobleaching and phototoxicity, which makes LSFM suitable for long-term observation of living specimens. Compared with point-scanning microscopy, wide-field detection in LSFM acquires images slice by slice with CMOS or CCD cameras, providing the advantage of rapid volumetric imaging. Owing to its outstanding performance, LSFM is currently widely applied in botany,^4^ developmental biology,^5^ cell biology,^6^ immunology,^7^ and neuroscience.^8^

In LSFM, the field of view and the axial resolution are determined by the shape and intensity distribution of the light sheet. For the conventional Gaussian light sheet, the axial resolution and FOV are mutually constrained. Specifically, a thinner light sheet enables higher axial resolution, yet at the expense of a reduced field of view. Although nondiffracting beams such as Bessel beams,^9^ Airy beams,^10^ and optical lattices^11^ can greatly extend the FOV in LSFM, their inherent side lobes generate substantial out-of-focus background, thereby compromising both axial resolution and imaging contrast. To address these issues, LSFM has been combined with other techniques such as structured illumination microscopy (SIM),^12^^–^^13^ stimulated emission depletion microscopy,^14^ and two-photon excitation microscopy.^15^^–^^16^ In our previous work, we have proposed and demonstrated the complementary beam subtraction (CBS) method,^17^^–^^19^ which achieves superior axial resolution alongside an expanded FOV by effectively eliminating the out-of-focus background.

Optical aberrations arising from sample-induced wavefront distortion and the imperfections of the optical components or alignment can degrade image quality and affect imaging depth in microscopy. Adaptive optics (AO), originally developed in astronomy to compensate wavefront distortions caused by atmospheric turbulence,^20^ constitutes a set of electro-optical devices and computational tools dedicated to optimizing the performance of optical systems.^21^ AO which involves aberrations measurement and aberrations correction has been applied in the illumination path^22^ or imaging path^23^ of LSFM. Furthermore, Liu et al.^24^ combined lattice light-sheet microscopy with aberrations correction in both illumination and detection paths by wavefront sensor-based adaptive optics. Such hardware solutions are typically complex and expensive to implement,^25^ restricting the broader applications of the technology. Liu et al.^26^^–^^27^ implemented sensorless AO in LSFM to correct aberrations induced by the electrically tunable lens, realizing nearly diffraction-limited fast volumetric imaging and improving the signal-to-background ratio by 3.5-fold.

In this paper, we employ a classic sensorless adaptive optics strategy, taking image sharpness as the metric and using Zernike modes as the basis for aberration representation. By quantifying the maximum metric values across different modes, we successfully implement aberration correction for the LSFM detection path. To further enhance imaging quality and axial resolution, we propose a method that integrates the sensorless adaptive optics with the complementary beam subtraction method, termed as AO-CBS. This approach acquires LSFM image stacks under two distinct illumination modes, i.e., Bessel beam light-sheet illumination and complementary Bessel beam light-sheet illumination, both corrected by AO. By subtracting the two image stacks, the final axial resolution is improved from 2.13±0.18  μm to 1.19±0.02  μm. To demonstrate the aberration correction capability of AO-CBS, we performed imaging experiments on fluorescent beads, *Aspergillus* conidia, cleared mouse liver section, and additional biological tissues.

## 2 Principle and Methods

**Fig. 1:**
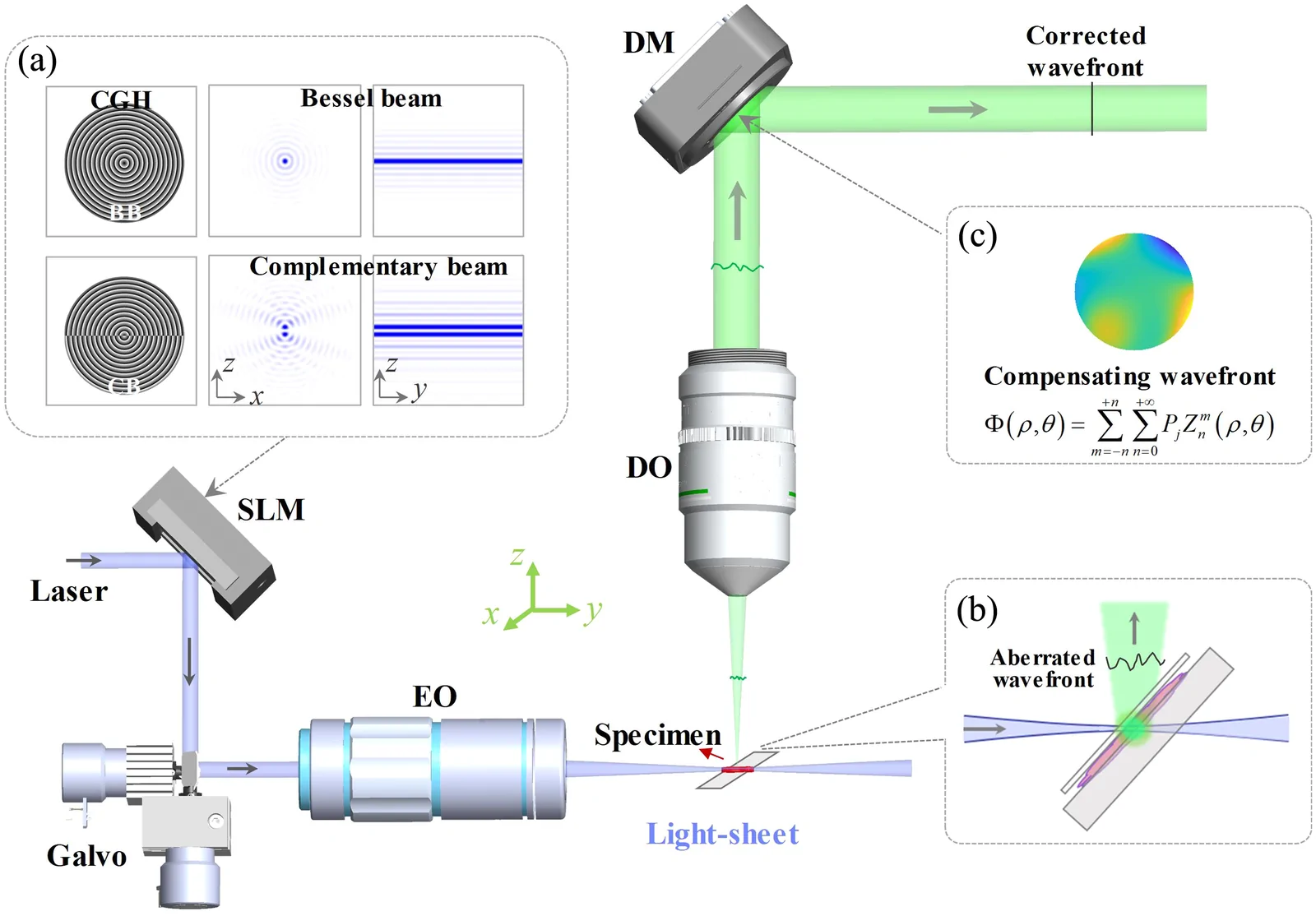
Schematic diagram of the proposed AO-CBS. Light sheets are generated by using a scanning galvanometer in the orthogonal optical path. The SLM in the illumination optical path generates nondiffracting beams, and the DM in the detection optical path achieves aberration correction. (a) The CGHs loaded on the SLM generate Bessel beam and its complementary beam, respectively. (b) Aberrations are introduced as light propagates through the specimen due to variations in refractive index. (c) The Zernike compensating wavefront, consisting of the finite Zernike modes, is loaded on the DM to correct aberrations.

The principle of the proposed AO-CBS method is illustrated in Fig. 1. The propagation-invariant Bessel beam $E_{\mathrm{BB}}(x,z)$ and its complementary beam $E_{\mathrm{CB}}(x,z)$ can be generated by loading computer-generated holograms (CGHs) onto the spatial light modulator (SLM), as shown in Fig. 1(a). Nondiffracting beams propagating along the y-axis are rapidly scanned along the x-axis with the Galvo mirror to generate light sheets. Thus, the Bessel light sheet is represented by $I_{\mathrm{BB}}(z)=\int |E_{\mathrm{BB}}(x,z){|}^{2}dx$ and the corresponding complementary light sheet is $I_{\mathrm{CB}}(z)=\int |E_{\mathrm{CB}}(x,z){|}^{2}dx$*.* As demonstrated in our previous study,^28^ the complementary beam subtraction method is equivalent to imaging under a novel effective light-sheet $I_{\mathrm{CBS}}(z)$ illumination, which can be represented as

$$I_{\mathrm{CBS}}(z)=I_{\mathrm{BB}}(z)-I_{\mathrm{CB}}(z).$$ (1)

Sample-induced wavefront distortion and the imperfections of the optical components or alignment result in an aberrated wavefront, as shown in Fig. 1(b). To correct the aberrations, we introduce the universal framework for sensorless adaptive optics.^29^ The distorted wavefront is decomposed into a set of finite and orthogonal Zernike modes. A deformable mirror conjugated to the rear pupil plane of the detection objective loads different Zernike modes with variable coefficients, and the coefficients of each Zernike mode are sequentially optimized using an image metric (e.g., sharpness or intensity). Specifically, a series of two-dimensional fluorescent images is captured with a digital camera for different values of the coefficient for each mode, and the corresponding image metrics are computed to determine the optimal coefficients. Prior to optimizing the next mode, the corresponding correction is applied to the wavefront via the deformable mirror, such that each successful mode correction enhances the signal for subsequent optimization steps. As shown in Fig. 1(c), the wavefront phase $\phi_{\mathrm{AO}}(k_{x},k_{y})$ after AO correction is described as

$$\varphi_{\mathrm{AO}}(k_{x},k_{y})=\underset{m,n}{\sum }\alpha_{n}^{m}Z_{n}^{m}(k_{x},k_{y}),$$ (2)

here, $\left(k_{x},k_{y}\right)$ is the coordinate in the rear pupil plane of the detection objective. Empirically, the aberrations induced by fluorescent beads and sectioned samples in the microscopes used here are well described by combination of finite Zernike modes. After correction, the electric field in the rear pupil of the detection objective for the image of a single punctum is expressed as

$$E_{\mathrm{AO}}(k_{x},k_{y})=P(k_{x},k_{y})\exp\{i[\phi_{\mathrm{Abb}}(k_{x},k_{y})-\phi_{\mathrm{AO}}(k_{x},k_{y})]\},$$ (3)

here, $P(k_{x},k_{y})$ is the pupil amplitude of the detection objective, and $\phi_{\mathrm{Abb}}(k_{x},k_{y})$ is the aberration phase. The 3D detection point spread function (PSF) after AO correction is approximated by

$${\mathrm{PSF}}_{\det}^{\mathrm{AO}}(x,y,z)=|\iint E_{\mathrm{AO}}\left(k_{x},k_{y}\right)\exp\left[i\left(k_{x}x+k_{y}y+k_{z}z\right)\right]dk_{x}dk_{y}{|}^{2}.$$ (4)

After correction, the detection PSF restores diffraction-limited performance.

For light-sheet fluorescence microscopy, the 3D overall PSF is

$${\mathrm{PSF}}_{\mathrm{overall}}^{\mathrm{AO}}(x,y,z)=I(z)\cdot {\mathrm{PSF}}_{\det}^{\mathrm{AO}}(x,y,z).$$ (5)

Here, I(z) is intensity distribution of the light sheet. The image stack of the AO-CBS is mathematically formulated in Eq. (6):

$${IM}_{\mathrm{CBS}}^{\mathrm{AO}}(x,y,z)={IM}_{\mathrm{BB}}^{\mathrm{AO}}(x,y,z)-{IM}_{\mathrm{CB}}^{\mathrm{AO}}(x,y,z)\;=S(x,y,z)*[I_{\mathrm{BB}}(z)\cdot {\mathrm{PSF}}_{\det}^{\mathrm{AO}}(x,y,z)-I_{\mathrm{CB}}(z)\cdot {\mathrm{PSF}}_{\det}^{\mathrm{AO}}(x,y,z)]\;=S(x,y,z)*\{I_{\mathrm{CBS}}(z)\cdot {\mathrm{PSF}}_{\det}^{\mathrm{AO}}(x,y,z)\}.$$ (6)

Here, S(x,y,z) represents the three-dimensional spatial distribution of fluorescent emitters in the specimen, and ∗ denotes the convolution operation.

**Fig. 2:**
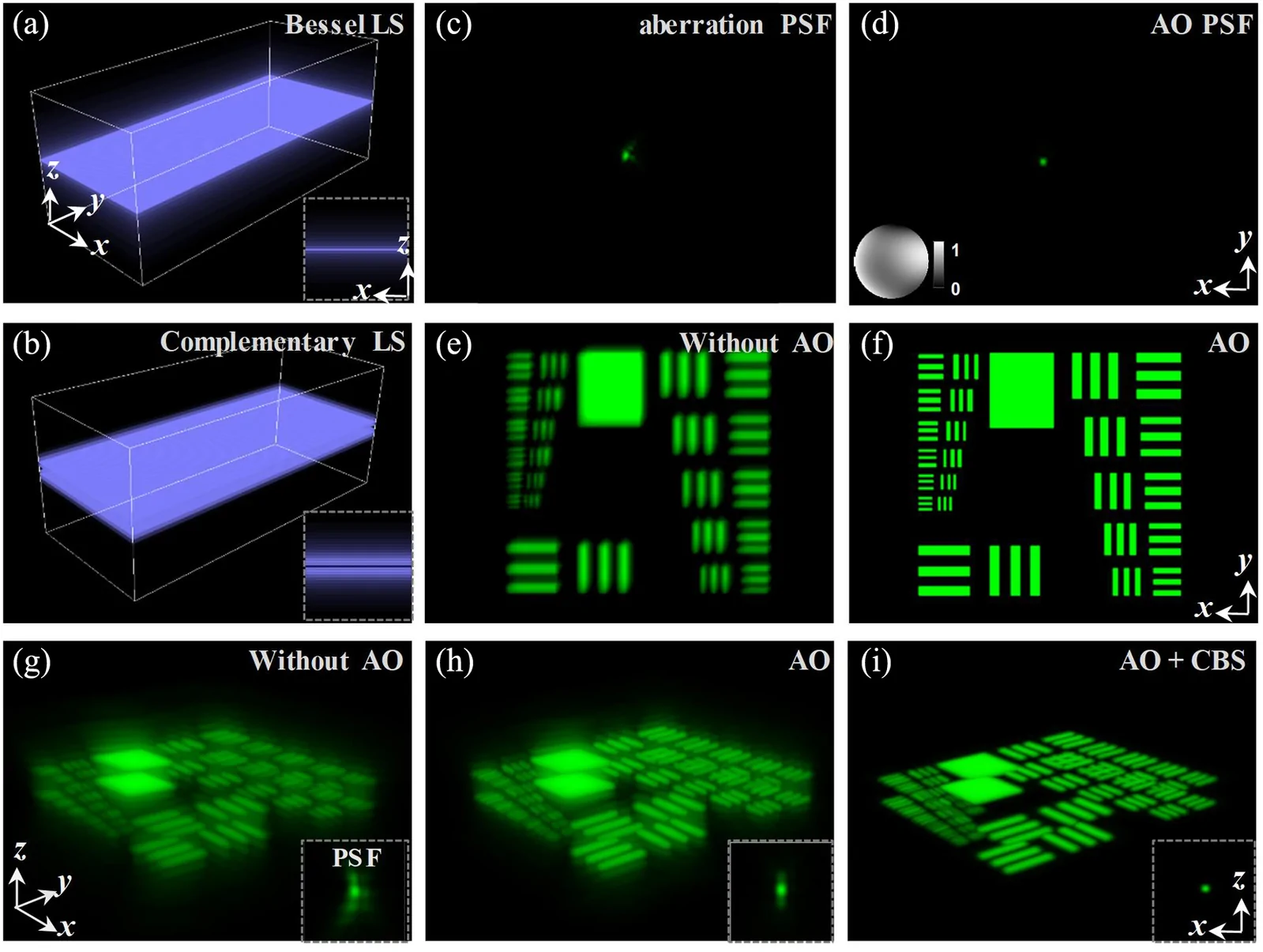
Numerical simulations of the proposed AO-CBS method. (a) and (b) LS patterns of the Bessel beam and the complementary beam, respectively. The insets show the intensity distribution of the LSs in the xz cross section. Beams propagating along the *y*-axis are scanned along the *x*-axis to generate LSs, and the detecting direction is along the *z*-axis. (c) and (d) PSFs in the detection path of LSFM with and without AO. The wavefront is shown with a normalized grayscale. (e) and (f) Simulated imaging results of the USAF 1951 resolution target with and without AO in the xy-plane. (g)–(i) Simulated 3D imaging results of the USAF 1951 resolution target with/without AO, as well as using the AO-CBS method, in the xy-plane. The insets show the different detection PSFs in the xz-plane.

Based on the above principle, we first implemented a numerical simulation, as shown in Fig. 2. Figures 2(a) and 2(b) present the 3D intensity distributions of the Bessel light sheet and the complementary beam light sheet, respectively. The diffuse and enlarged detection PSF [Fig. 2(c)] is restored to a diffraction-limited PSF as depicted in Fig. 2(d) after AO correction, which results in the blurred and dim image [Fig. 2(e)] becoming brighter and clearer [Fig. 2(f)]. Two layers of USAF 1951 resolution targets, parallel to the xy-plane and separated by a certain distance along the z-axis, are selected as our three-dimensional samples. The convolution of these samples with the corresponding 3D overall PSF yields the 3D imaging results in LSFM. The simulated imaging results and the 3D overall PSF both indicate that AO enhances image quality and signal intensity, though the axial resolution shows no significant improvement, as depicted in Fig. 2(h). The combination of AO and complementary beam subtraction not only improves the imaging quality but also increases the axial resolution, as shown in Fig. 2(i). The simulation further validates the effectiveness of the AO-CBS method.

## 3 Experimental System

**Fig. 3:**
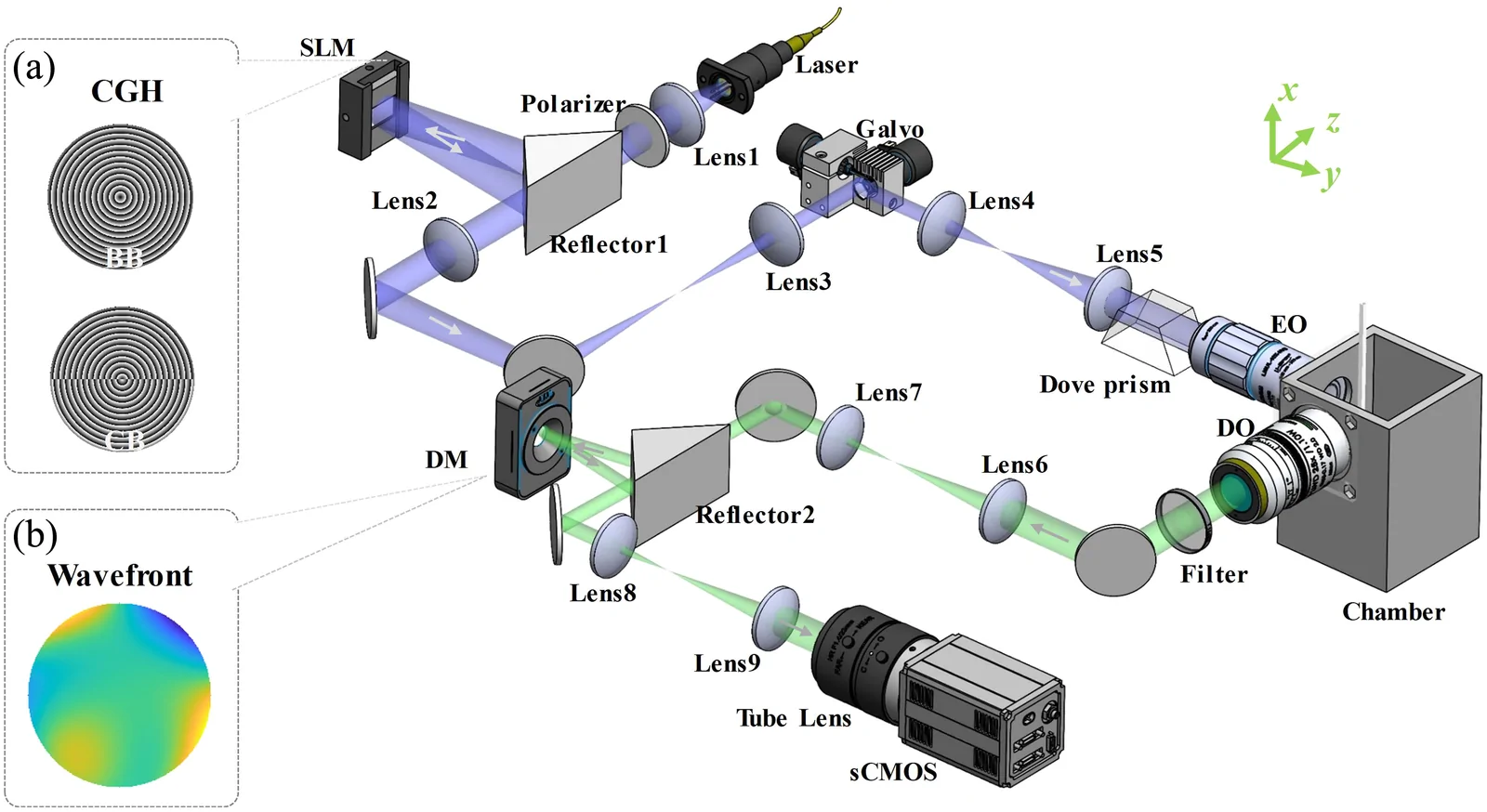
Schematic diagram of the LSFM system. SLM, spatial light modulator; DM, deformable mirror; EO, excitation objective; DO, detection objective. (a) The CGHs loaded on the SLM can, respectively, generate the Bessel beam and its complementary beam. (b) The wavefront loaded on the DM after AO correction.

To experimentally validate the method, we developed a light-sheet fluorescence microscopy system, with the schematic diagram depicted in Fig. 3. In the illumination path, the excitation light is delivered via a single mode fiber from cw lasers (Lambda beam fiber 488-90 SM, RGB Photonics Inc., Germany), is first collimated with a lens (f1=50  mm) and then transmitted through a linear polarizer to generate a horizontally linear polarized beam. After reflection from the first triangular reflector, the linearly polarized beam is incident on a phase-only spatial light modulator (SLM, PLUTO-2-NIR011, Holoeye Inc., Germany). By loading the CGHs [Fig. 3(a)] onto the SLM, Bessel beams and the complementary beams are generated, respectively, and subsequently conjugated to a galvanometric scanning mirror (GVSM002/M, Thorlabs Inc., USA) via a pair of relay lenses (f2=300  mm, f3=100  mm). The light sheet, produced by scanning the galvanometric scanning mirror along the x-axis, is expanded 3.2× using a Galilean type beam expander (f4=39  mm, f5=125  mm) and conjugated to the rear pupil of the excitation objective (10× NA 0.45 WD 19 mm, 58-372, Edmund Inc., USA). Specimens held in glass capillaries or mounted on glass slides are illuminated with the light sheet in the custom-designed, water-filled 3D-printed imaging chamber. In the detection path, the fluorescent signals from the specimens are collected by the detection objective (water dipping, NA 1.1 WD 2 mm, CFI Apo 25× W NIR, Nikon Inc., Japan), and then pass through a quad-notch filter (NF03-405/488/532/635 E25, Semrock Inc., USA). The beam diameter of the fluorescent signal is de-magnified by a factor 2.7× via a beam compressor (f6=400  mm, f7=150  mm). The fluorescent signal is then reflected by the second triangular reflector onto a deformable mirror (DM, 97-15, Alpao Inc., France), which is relayed to the rear pupil plane of the detection objective. After aberration correction with the DM, the fluorescence signal is imaged onto the digital camera (ORCA-Lightning, Hamamatsu Inc., Japan) through another pair of relay lenses (f8=150  mm, f9=180  mm) and a zoom lens (focal length=75 to 300 mm, Nikon, Japan). The effective magnification of the LSFM is ∼55×, yielding ∼100  nm per pixel.

The sample was illuminated with a Bessel light sheet during the aberration correction process. The DM loads 6 modes of the first 10 Zernike modes with variable coefficients, excluding piston ($Z_{0}^{0}$), tip ($Z_{1}^{-1}$), tilt ($Z_{1}^{1}$), and defocus ($Z_{2}^{0}$). The coefficients of each Zernike mode are divided into a range of steps from −1 to 1, thus a range of image frames is captured with the sCMOS camera. Image sharpness (i.e., standard deviation of image gray values) is adopted as the metric, and multiple values of image metrics are computed to obtain the optimal coefficient values for the corresponding modes. For sparse samples such as fluorescent beads, optimal coefficients can be obtained via measuring the metric of a few steps as quadratic functions. In the case of dense samples like *Aspergillus* conidia, where quadratic function is often ineffective, we commonly resort to 21 coefficient steps. Prior to optimizing the subsequent mode, the corresponding correction is applied to the wavefront generated by the DM. This ensures that each successful correction of a mode enhances the signal intensity for the next mode optimization. The time for a correction cycle takes about 13 s. The AO correction time is affected by various factors, such as the frame rate of the sCMOS camera, the refresh rate of the DM, and the AO metric computational time. Figure 3(b) shows a schematic diagram of the Zernike mode optimized wavefront loaded on the DM. After AO correction, the samples are alternately illuminated by the Bessel light sheet and its complementary Bessel light sheet, and two images are obtained for each axial position. Three-dimensional volumetric imaging is achieved by axially translating the sample across the LS using a motorized translation stage (MTS25-Z8, Thorlabs Inc., USA) and two image stacks are captured by the sCMOS camera. The CBS results are obtained by subtracting the two image stacks. The imaging speed of the CBS method is influenced by various factors, e.g., the moving speed of the motorized translation stage, the frame rate of the camera, and the refresh rate of the SLM. The current bottleneck of imaging speed in our system is the SLM. A faster SLM will significantly increase the imaging speed.

## 4 Results and Discussion

**Fig. 4:**
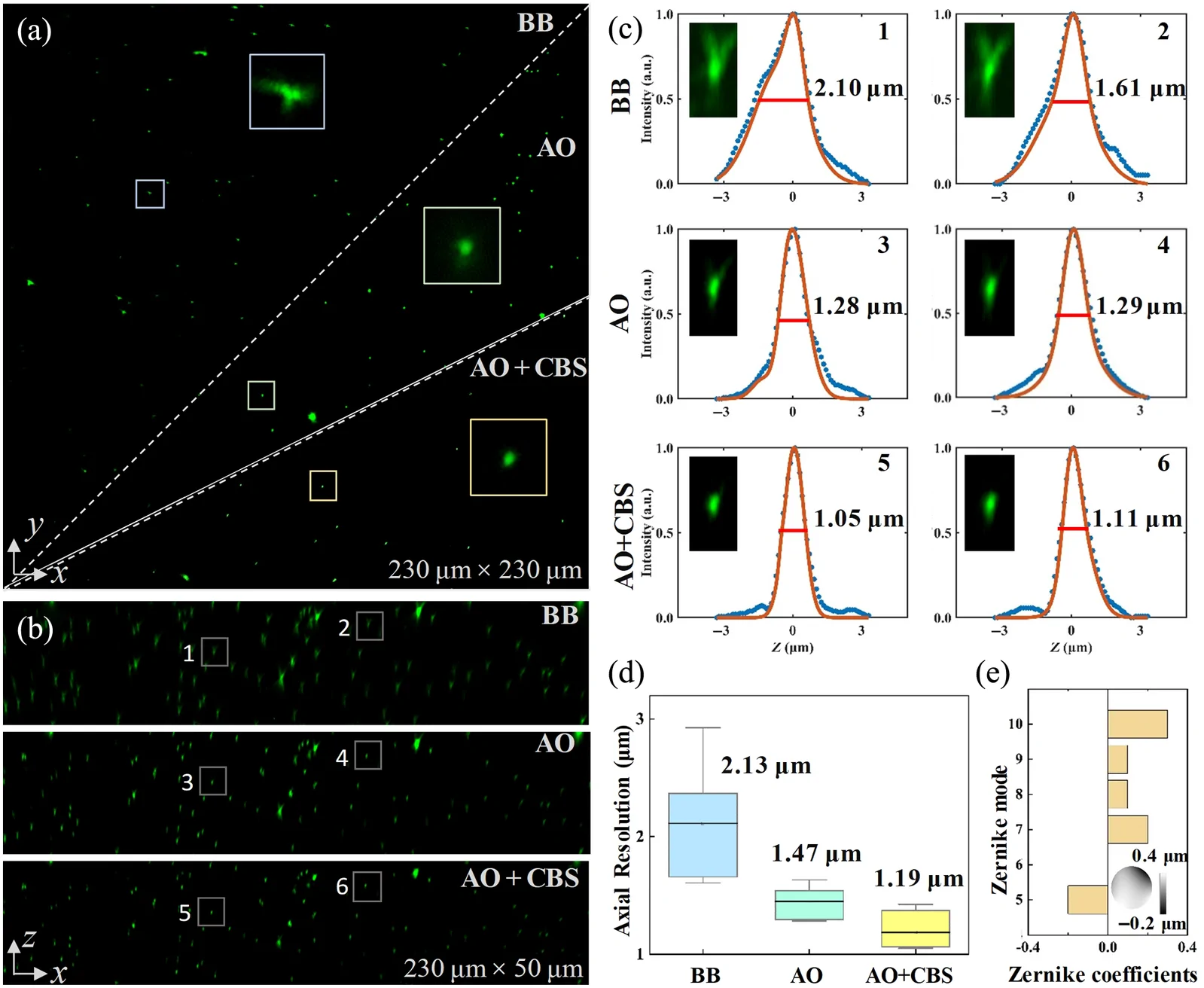
Imaging results of 0.5  μm-diameter fluorescent beads. (a) and (b) The *xy*-plane and *xz*-plane MIP images of fluorescent beads excited by BB LS before/after AO correction, and the AO-CBS method, respectively. (c) The normalized intensity profiles along the z-axis of the fluorescent beads selected in panel (b). The insets are the magnified views of the selected beads indicated by the dashed box in panel (b). (d) Box plots of the full width at half maximum of 10 fluorescent beads under different methods. (e) The bar plot depicts the Zernike decomposition of the estimated aberrations for each setting. The inset shows the wavefront on the DM after AO correction.

We first evaluated the AO-CBS method by imaging yellow-green fluorescent beads (0.5  μm in diameter, 505/515, Invitrogen, USA) embedded in agarose gel. The imaging results of the AO-CBS method were obtained by subtracting the two image stacks (100 slices, axial interval 0.5  μm, 2304×2304  pixels, 230×230×50  μm) acquired after AO correction, with one illuminated by the Bessel beam light sheet and the other by its complementary Bessel light sheet. The maximum intensity projection (MIP) images obtained by illuminating the fluorescent beads with Bessel beam LS before/after AO correction, and the AO-CBS method are shown in Fig. 4, respectively. The maximum gray level of fluorescent beads image is increased from 2616 to 3867 after AO correction, resulting in a notable enhancement of signal intensity by 48%. To quantify the axial resolution, the full width at half maximum (FWHM) was measured on ten fluorescent beads, with the statistical average value adopted as the axial resolution. The results for the beads are shown in Fig. 4(c). The statistical axial FWHMs are 2.13±0.18  μm for Bessel beam (BB) LS, 1.47±0.02  μm for BB LS after AO, and 1.19±0.02  μm for the AO-CBS method, as shown in Fig. 4(d). The results demonstrate that both the axial resolution and image contrast are significantly enhanced by the AO-CBS method. The bar plots in Fig. 4(e) depict the Zernike decomposition of the estimated aberrations for each setting.

**Fig. 5:**
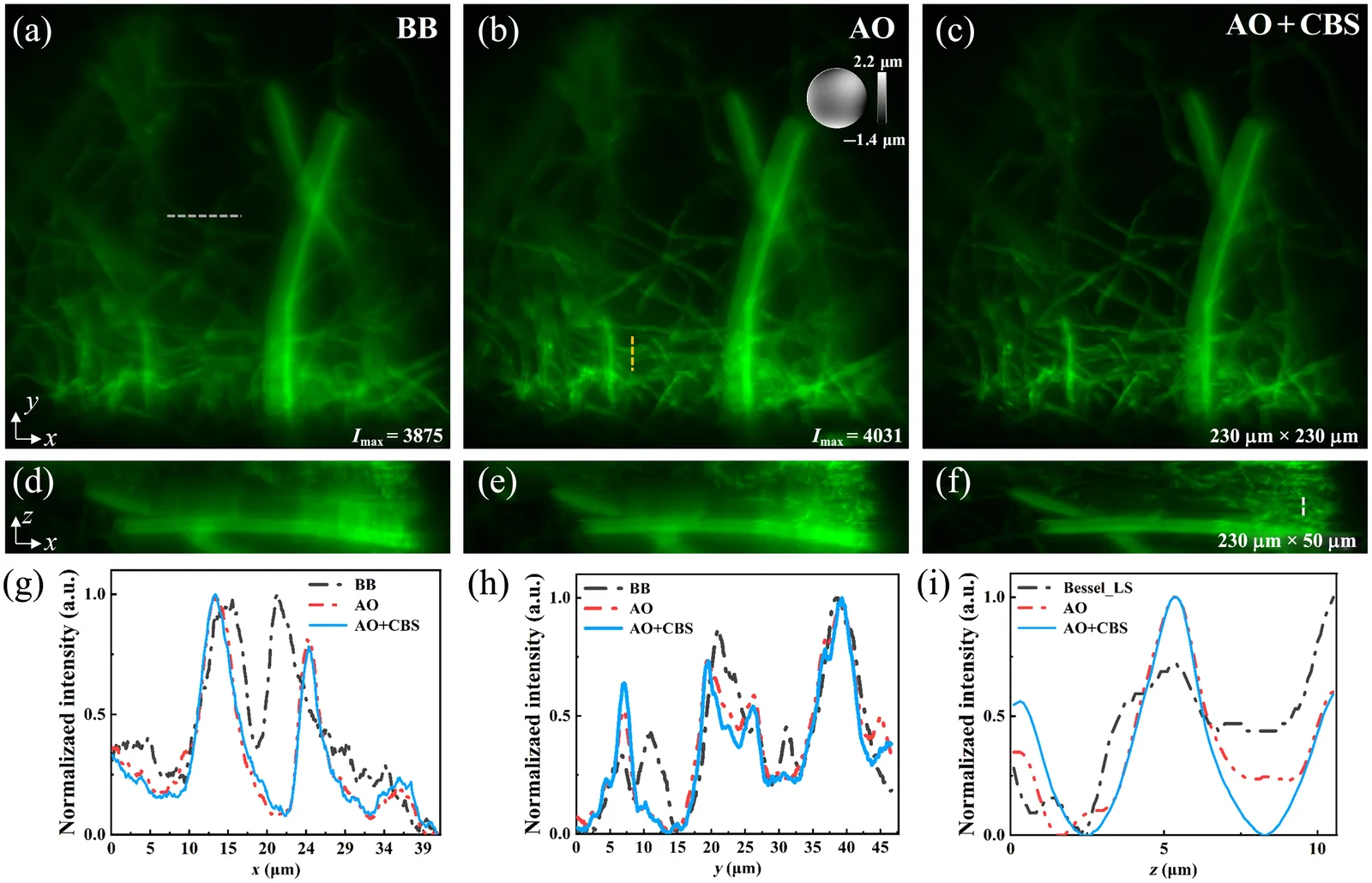
Imaging results of *Aspergillus* conidiophores excited by 532 nm laser. (a)–(c) The xy-plane MIP images of the sample by using BB LS before/after AO correction, and the AO-CBS method, respectively. (d)–(f)  The xz-plane MIP images of the sample by the three methods, respectively. (g)–(i)  The normalized intensity profiles along the dotted lines in panels (a), (b), and (f), respectively.

Subsequently, imaging experiments were performed on the *Aspergillus* conidiophores (Carolina Inc., USA) to compare the imaging performance of the AO-CBS methods. Each stack of raw images contains 50 slices @ 2304×2304  pixels with an axial interval of 1  μm, and the FOV is 230×230  μm. The MIP images in the xy-plane and the xz-plane are shown in Figs. 5(a)–5(f), which are obtained with Bessel beam LS before/after AO correction, and the AO-CBS method, respectively. The images under the BB LS illumination with AO correction provide better resolution compared with the images illuminated by BB LS. The subtraction method further effectively eliminates the out-of-focus background, thus significantly improving the axial resolution. The normalized intensity profiles along the x-axis, y-axis, and z-axis, corresponding to the gray dotted lines in Fig. 5(a), yellow dashed lines in Fig. 5(b), and white dashed lines in Fig. 5(f), respectively, are presented in Figs. 5(g)–5(i). These profiles further demonstrate the superior imaging performance of the AO-CBS method.

**Fig. 6:**
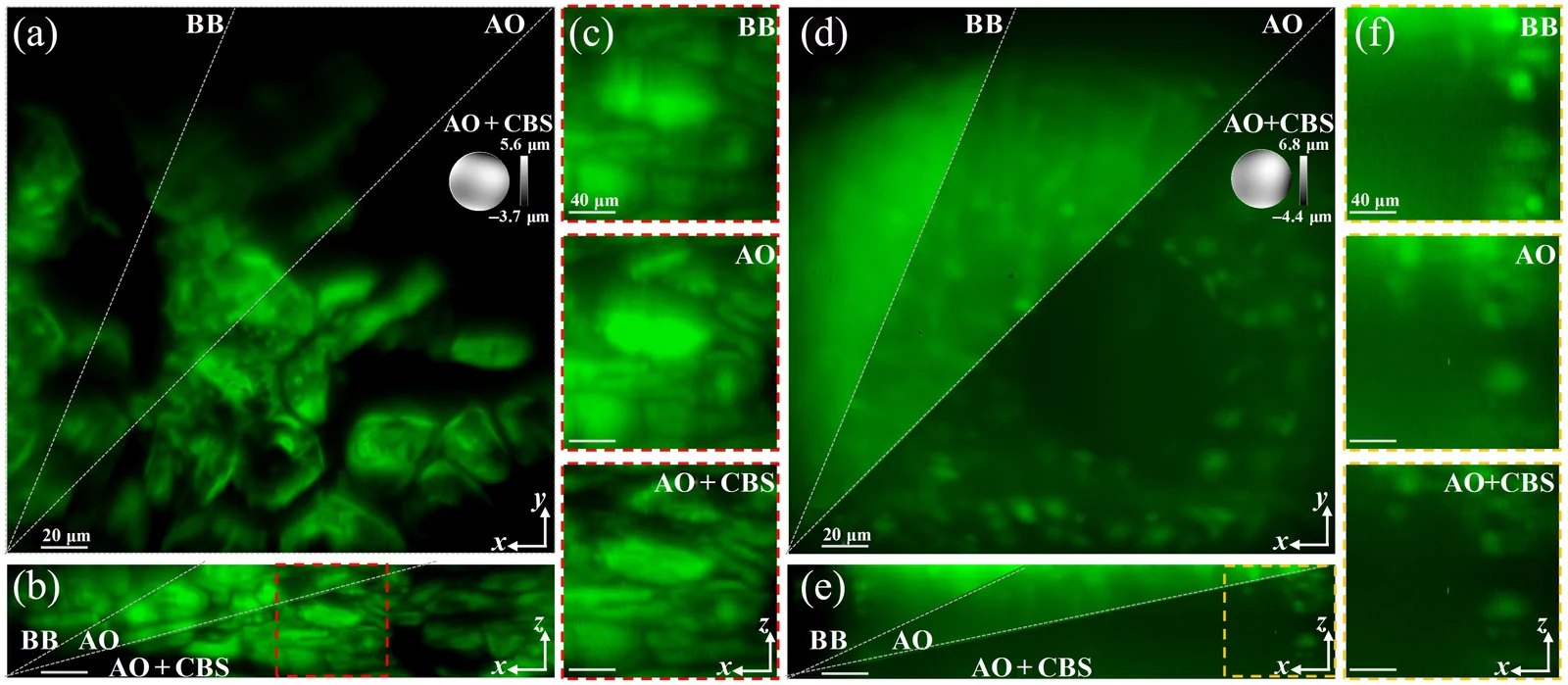
Imaging results of the pear fruit slide and cleared mouse liver section excited by 532 nm laser. (a) and (b) The xy-plane and xz-plane MIP images of the pear fruit sliced sample by using the BB LS before/after AO correction, and the AO-CBS method, respectively. (c) The zoom-in parts of the marked areas indicated in panel (b). (d) and (e) The xy-plane and xz-plane MIP images of cleared mouse liver section by using the three methods, respectively. (f) The zoom-in parts of the marked areas indicated in panel (e).

To further validate the imaging performance, we performed the imaging experiments on the pear fruit slide (Xinxiang Red Forest Edu. Instrument Co., Ltd., China) and cleared mouse liver section (PS004, SunJin Lab Co., China). Each raw image stack consists of 50 slices, each acquired with the image of 2304×2304  pixels and an axial interval of 1  μm. The MIP images in the xy-plane and the xz-plane are shown in Figs. 6(a) and 6(b) and 6(d) and 6(e), which are obtained with the BB LS illumination, the BB LS illumination after AO correction and the AO-CBS method, respectively. The superior imaging performance of the AO-CBS method is further demonstrated by the locally magnified views of the three imaging results in the xz-plane along the dashed box in Fig. 6(b), as presented in Fig. 6(c). As demonstrated in Fig. 6(f), this method delivers identical imaging quality for animal samples as it does for plant samples. In our research, we correct the wavefront distortion in the detection path of LSFM, while the CBS method was employed to enhance the tomographic capability of optical sections, thereby improving axial resolution.

Compared with sensor AO, sensorless AO is easy to be implemented without additional adaptive correction elements. Because there is no separate aberration sensing path to suffer from the non-common-path aberration,^30^ aberrations arising from both the optical system and the specimen can be sensed by the sensorless AO method. However, the sensorless AO takes much time to correct optical aberrations. This drawback may be addressed by combining the deep learning approach with the sensorless AO in LSFM.^31^ There is no wavefront sensor in the LSFM system, and therefore the rms value of the residual aberrations cannot be measured directly after AO correction. The rms value of the corrected wavefront was calculated, which indicates that the aberrations in biological samples are larger than those in fluorescent beads. The AO correction method improved image brightness and image contrast, for either fluorescent beads or biological samples, which confirms the actual performance of the AO on all these samples.

## 5 Conclusion

In summary, we have proposed an adaptive optics with complementary beam subtraction (AO-CBS) method to compensate wavefront distortions and enhance the axial resolution in LSFM. By subtracting the image stacks obtained from the Bessel beam scanning and its complementary beam scanning, the out-of-focus background interference is effectively eliminated, thereby enabling a substantial improvement in axial resolution. This method effectively addresses key issues existing in traditional Bessel beam-based LSFM, including the out-of-focus background induced by sidelobes, the limited axial resolution, and the degraded image quality caused by optical aberrations. Both the simulation and the imaging experiments on fluorescent beads and biological tissues consistently demonstrated superior performance in suppressing out-of-focus background signal and enhancing axial resolution. The idea of combining adaptive optics with complementary beam subtraction method in LSFM can serve as a design strategy to upgrade the light-sheet-based imaging systems and drive the further development and broader applications of LSFM.

## Data Availability

Data will be made available on request.
